# Analysis of *Giardia lamblia* Nucleolus as Drug Target: A Review

**DOI:** 10.3390/ph16081168

**Published:** 2023-08-16

**Authors:** Carlos Gaona-López, Ana Verónica Martínez-Vázquez, Juan Carlos Villalobos-Rocha, Karina Janett Juárez-Rendón, Gildardo Rivera

**Affiliations:** 1 Laboratorio de Biotecnología Farmacéutica, Centro de Biotecnología Genómica, Instituto Politécnico Nacional, Reynosa 88710, Mexico; 2 Centro de Biotecnología Genómica, Instituto Politécnico Nacional, Reynosa 88710, Mexico; avmartinez@ipn.mx (A.V.M.-V.); kjuarezr@ipn.mx (K.J.J.-R.); 3 Departamento de Microbiología, Escuela Nacional de Ciencias Biológicas, Instituto Politécnico Nacional, Ciudad de México 11340, Mexico; jvillalobosr@ipn.mx

**Keywords:** *Giardia lamblia*, nucleolus, target, drugs, antiprotozoal

## Abstract

*Giardia lamblia* (*G. lamblia*) is the main causative agent of diarrhea worldwide, affecting children and adults alike; in the former, it can be lethal, and in the latter a strong cause of morbidity. Despite being considered a predominant disease in low-income and developing countries, current migratory flows have caused an increase in giardiasis cases in high-income countries. Currently, there is a wide variety of chemotherapeutic treatments to combat this parasitosis, most of which have potentially serious side effects, such as genotoxic, carcinogenic, and teratogenic. The necessity to create novel treatments and discover new therapeutic targets to fight against this illness is evident. The current review centers around the controversial nucleolus of *G. lamblia*, providing a historical perspective that traces its apparent absence to the present evidence supporting its existence as a subnuclear compartment in this organism. Additionally, possible examples of ncRNAs and proteins ubiquitous to the nucleolus that can be used as targets of different therapeutic strategies are discussed. Finally, some examples of drugs under research that could be effective against *G. lamblia* are described.

## 1. Introduction

*Giardia lamblia* (*G. lamblia*), syns. *Giardia intestinalis* (*G. intestinalis*) or *Giardia duodenalis* (*G. duodenalis*) is a unicellular protist parasite, the etiological agent of giardiasis, which is considered one of the main causes of diarrhea worldwide. The World Health Organization (WHO) reports that this parasitosis more severely affects children under five years of age and that pneumonia and diarrheal diseases, including giardiasis, are the most common causes of infant death in this age group, representing 13% and 9%, respectively in 2015 [1,2]. Additionally, intestinal parasites are an important source of morbidity among the adult population, creating a significant demand for healthcare services. Giardiasis was added to the WHO’s Neglected Diseases Initiative in 2004 [3]. Finally, this disease is characterized by negatively affecting the physical and mental development of individuals with chronic giardiasis in the early stages of development, preventing them from irreversibly developing their full potential [4,5].

Giardiasis is a disease with a strong socioeconomic component, denoted by its incidence. In low- and middle-income countries, 20% to 30% of the population is infected, while high-income countries report an incidence of between 2% and 5% of their population [6]. In both cases, the disease mostly affects children who have poor hygiene habits. Additionally, individuals with compromised immunity are more susceptible to suffering from severe and long-lasting infections [6,7]. Interestingly, *G. lamblia* was considered a commensal organism of the human digestive tract until 1981, when the WHO recognized *Giardia* as a pathogenic organism [8,9].

As mentioned earlier, this parasite infection is unevenly distributed and characterized by a prominent socioeconomic factor. While in the United States of America (USA) and Europe, just over 10,000 cases are reported annually, mainly associated with isolated outbreaks among travelers visiting countries where the disease is most prevalent [10,11]. In contrast, Latin America, Asia, and Africa report more than two hundred million cases during the same period [10,11].

Currently, there are various drugs to combat this disease, those being nitroimidazole derivative compounds (Table 1), the most widely used as first-line drugs [11,12]. Benzoylmetronidazole, ornidazole, tinidazole, and metronidazole are among the compounds in this class; the latter is preferred for the treatment of giardiasis (Table 1). Regrettably, studies have shown that the various nitroimidazole derivative compounds used to treat giardiasis may have genotoxic or even carcinogenic effects in animal models to a therapeutic dose, and the potential impacts on human health are still a topic of debate [12,13,14,15]. Another group of widely used medications for treating giardiasis are broad-spectrum benzimidazoles, including albendazole and mebendazole (Table 1). However, the use of these drugs during pregnancy is not recommended due to their proven teratogenic effects in animals and possible effects in humans [12,14,16,17]. It should be highlighted that mebendazole as a teratogen in humans is still being discussed. Another widely used drug is furazolidone (Table 1), which is broad-spectrum, inhibiting the growth of Gram-negative and Gram-positive bacteria alike [12]. It is crucial to point out that for several years this drug has been considered a trigger for neoplastic processes and genotoxic by the Food and Drug Administration (FDA), at least in animals. As a result, its use has been prohibited in the USA [12,18]. Finally, other option is nitazoxanide (Table 1), a broad-spectrum antimicrobial, which was approved for use in the USA in 2003 by the FDA [19]; currently, no serious adverse effects such as those mentioned above have been documented, delimiting the drawbacks mainly to nausea, abdominal pain, pruritus, all considered mild [20,21]. Since the rest of the antiparasitics date from the second half of the last century, the need to develop new drugs to combat this parasitosis is imperative, coupled with the fact of the growing resistance to the drugs used, as well as the low therapeutic adherence to the treatment due to adverse effects [12,20].

Giardiasis has been considered a neglected disease, predominant in underdeveloped countries; however, the migratory flows due to poverty, wars, and lack of opportunities have led to the spread of this parasitosis from regions where it is common to places where it was not [29,30]. Because these diseases are characteristic of low- to middle-income countries, the development of new drugs and the search for therapeutic targets have been under-researched, mainly due to the social dilemma that the market does not guarantee a strong economic return for research [31].

According to Müller et al., 2016, there are two main strategies for the identification of therapeutic targets in parasites. On the one hand, we have whole organism screening, approaching from which most antiparasitic drugs have been obtained. On the other hand, target-based drug design, in theory, shows great potential, as demonstrated in other groups of protists, where it has been established that identifying the appropriate therapeutic target is a crucial aspect of effective treatment [31,32].

In this review, we intend to summarize those therapeutic targets within the nucleolus of the intestinal parasite *G. lamblia*. For this purpose, a thorough bibliographic search was carried out in the different repositories of specialized scientific literature, using the nucleolus of the *G. lamblia* parasite as search criteria.

## 2. Cell Biology of *G. lamblia*

**Figure 1:**
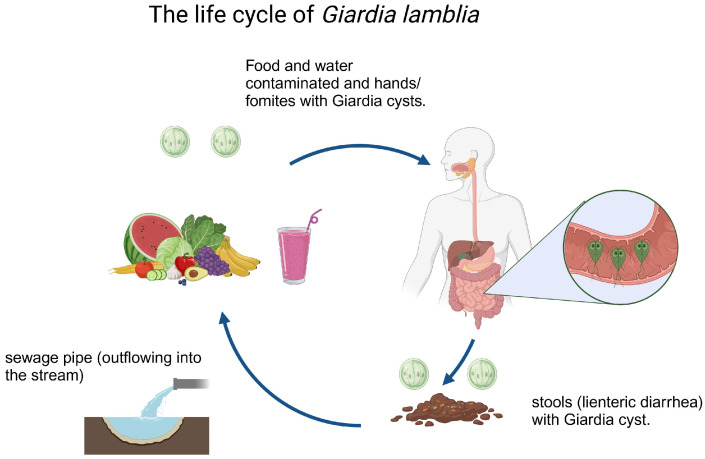
The life cycle of *G. lamblia* comprises two phases: the trophozoite, an active form that attaches and reproduces in the host’s small intestine, and the cyst, a dormant form shed in the feces that infects new hosts. Transmission occurs through ingestion of contaminated cysts present in water or food.

*G. lamblia* has a simple life cycle (Figure 1), which consists of two stages: the trophozoite, which is considered the vegetative and replicative form, which usually adheres to the epithelial cells of the anterior part of the small intestine [33]; an important aspect to highlight is that this stage is the target of all chemotherapeutic therapies. On the other hand, the cyst is the infective form and is considered the latent form that interacts with the environment. Studies have indicated that the cyst can remain viable for weeks or even months at warm temperatures and is resistant to chlorine concentrations typically used for water purification [34,35]. Likewise, the trophozoite presents a binucleate morphology, while the cyst is tetranucleate [36]. This parasite presents a fine-tuned regulated life cycle, whose main form of reproduction is asexual, through binary fission, and its sexual reproduction is currently the subject of great research, which has been speculated would contribute to genetic variability, essential for the acquisition of resistance to antibiotics [36,37].

## 3. The Genome of *G. lamblia*

The analysis of the genome indicates that *G. lamblia* presents a relatively compact genome with respect to other eukaryotes, with approximately 12 Mb in size distributed in 5 chromosomes, presenting almost 4800 expressed genes, which was determined by the Serial Analysis of Gene Expression (SAGE) technique [38,39]. It is important to note that, based on the analysis of *Giardia’s* genome, no homologous enzymes of the Krebs cycle have been reported. Additionally, there is a smaller number of enzymes responsible for the biosynthesis of amino acids and for synthesizing and metabolizing lipids. This is primarily due to *Giardia’s* parasitic lifestyle, as it relies on obtaining these molecules from its host rather than producing them internally [38,40,41]. Moreover, it was observed that there is a limited number of introns and mitochondrial genes, denoting a compact genome in size and number of genes [38,42]. Lastly, a rudimentary transcription machinery for all three sets of RNA polymerases has been reported [38,43].

## 4. Nucleolus of *G. lamblia*

The initial hypothesis that *G. lamblia* lacked a nucleolus was disproven by several cell biology studies [44], which provided increasing evidence of the existence of a small nucleolus located in the anterior region of each of this parasite’s nuclei. Such a structure would contain a reduced and rudimentary polRNAI transcription system, where various of their components have been described as highly divergent, which could have an important implication in the transcription processes, as well as ribosomal subunit assembly and ribosome production [43]. As a result of these studies, it was hypothesized that *G. lamblia* exhibits a variety of transcription initiation features that are both distinctive and characteristic of this parasite [43] It is important to point out that despite the *Giardia* nucleolus being initially thought to be one of the smallest and most minimalist known, current ultrastructural and proteomic information suggests the existence of a small nucleolus in archaea, a structure considered unique to eukaryotes [45].

### 4.1. The Minimalist Nucleolus of G. lamblia as a Potential Therapeutic Target

The existence of the *Giardia* nucleolus has been a subject of controversy, which stemmed from the observations made in 1998 regarding the expression and cytolocalization of the fibrillarin protein in *Giardia*. In that study, the signal for the protein was scattered throughout the nucleus, unlike in other eukaryotes, where it is restricted to a specific compartment in the nucleolus [46,47,48]. The study found that while the core of the enzyme is conserved in *Giardia*, the glycine and arginine residues (GAR domain) located at the amino terminus has a high content of aspartate (Asp) and proline (Pro) residues, with eleven of each compared to one and two, respectively, in the human protein. The authors report that the eleven Asp residues and one glutamate (Glu) residue are unique to *Giardia*, while the eleven Pro residues are shared only with one other protist, *Tetrahymena thermophila* [46,49,50]. In this sense, the information suggests that despite the degree of core conservation between *Giardia* and human fibrillarin, the use of RNA interference (RNAi) or CRISPR interference (CRISPRi) therapy could take advantage of the high specificity of the techniques to inhibit the expression of this gene in *Giardia* without apparent side effects or off-targets, taking into consideration that the terminal amino domain is sufficiently divergent for this type of therapy. Recalling that fibrillarin is essential for rRNA processing and maturation [48]. It should be noted that even though several early divergent protists lack an RNAi pathway, the presence of such machinery has been reported in *Giardia* [51]. In addition, it was initially determined through specific contrast techniques that the nucleolar organizer regions (NORs) did not appear to exist. Suggesting that ribosomal DNA (rDNA) transcription was homogeneous within the *Giardia* nucleus, which was regarded as a hallmark of a primitive state [52].

Afterward, a considerable amount of evidence emerged supporting the existence of a nucleolus in *Giardia*, contradicting the initial determination that the NORs did not appear to exist. First, there is evidence of the existence of proteins in *G. lamblia* that are ubiquitous to the nucleolus, such as the krr1 protein, which is considered essential for the synthesis and assembly of the 40S small ribosomal subunit [53,54]. This protein presents a low identity regarding its homolog in yeast (41.62%) [54]. Similarly, to fibrillarin, the krr1 gene and mRNA could be susceptible to being a therapeutic target through RNAi or CRISPRi therapy, compromising ribosomal synthesis to the detriment of the parasite’s viability. Additionally, it has been reported that the krr1 gene in *Giardia canis* (*G. canis*) plays an important role in the growth of the parasite, which was confirmed by the knock-down of the gene by hammerhead ribozyme flanked by krr1 antisense RNA [55]. It is worth noting that the authors do not report the identity percentage regarding the human ortholog protein, which is expected to be lower. Likewise, they consider the presence of krr1 as an indication that *Giardia* anucleolysis is a secondary condition in evolutionary terms [54].

The existence of a nucleolar compartment in *G. lamblia* is further supported by the identification of small nucleolar RNAs (snoRNAs), which are responsible for carrying out chemical modifications in the rRNA, such as methylation and pseudouridylation [56]. These changes are responsible for the structural and functional maturation of rRNA [56].

The first report on small nucleolar RNAs (snoRNAs) in *Giardia* was from Yang et al., 2005 [57]. Through the construction of a cDNA library, they reported the existence of 20 possible snoRNAs, which present the characteristics boxes C/D or H/ACA sRNAs (small RNAs that play a key role in ribosome biogenesis), as well as complementarity with the 16S-like rRNA subunit of *Giardia*, responsible for the aforementioned chemical modifications [56]. It is worth mentioning that of the 20 snoRNAs reported, only the location of *Gl*sR17 was known using the fluorescence in situ hybridization technique (FISH) [58], which locates this snoRNA in the anteronuclear region of *Giardia*. Additionally, these authors report that counterparts of almost 20 snoRNAs have been found in other eukaryotes, except for *Gl*sR3, *Gl*sR9, *Gl*sR10, and *Gl*sR11 [58]. The latter apparently do not have homologs in other eukaryotes, which present a perfect complementarity with the 16S-like rRNA subunit of *Giardia* [58], suggesting a role in its maturation. This information suggests that the exclusivity of these four snoRNAs to *Giardia* makes them a potential target for therapeutic intervention through gene silencing tools such as CRISPRi or RNAi without the risk of off-target effects.

A precedent on the use of these techniques for the silencing of snoRNAs is found in small nucleolar RNA 42 (SNORA42) in human lung cancer. In this study, through gain/loss-of-function experiments, the authors were able to inhibit tumorigenicity in lung cancer cells (NSCLC cells), as well as modulate cell proliferation and growth in both bronchial epithelial cells and NSCLC cells using the RNAi technique [59,60]. Additionally, in a study conducted by Siprashvili et al., 2016 [61], the deletion of two snoRNAs in various types of cancer was reported. Through their experiments, they were able to enhance tumorigenicity in murine models by deleting SNORD50A/B using CRISPR/Cas9, which had an inhibitory effect on the Ras oncoprotein (responsible for modulating signaling pathways), promoting the uncontrolled growth of tumor cells [60,61,62]. Finally, the first studies on the modulation of gene expression in *Giardia* using a derivative of this technique, CRISPRi, have been carried out, demonstrating the potential of this approach as a therapeutic resource [63,64]. In these experiments, two distinct transcriptional repression systems were designed independently to selectively inhibit the expression of specific genes, thus compromising parasite viability [63,64].

In 2011, was reported the first crystallographic structure of the *Giardia* snoRNA-bound ribonucleoprotein (snoRNPs). This complex is responsible for modifying rRNA. These authors report both structural and functional characteristics of both archaea and eukaryotes, concluding that the snoRNP protein element (15.5 kD Box C/D) occupies a unique evolutionary position between both domains of life (eukarya and archaea) [65]. This data suggest that the analysis of structure could provide guidance for the development of drugs that potentially target the archaean characteristics of this protein, leaving the ortholog protein in the host free of side effects. Using a target-based drug design could exploit the differences between both proteins.

Additionally, two possible eIF4E transcription initiation factors have been identified in *Giardia*, called eIF4E1 and eIF4E2. Of these two factors, only the latter appears to play a role in recognizing the 5′ cap, while eIF4E1 is located in a discrete region of the *Giardia* nucleus. The authors suggest that eIF4E1 is very possibly a snoRNA-binding protein, i.e., a snoRNP, speculating that it plays a role in rRNA and ribosome maturation [66].

Finally, Lagunas Rangel et al. reported the role of a sirtuin (GdSir2.4) in the modulation of rRNA expression in the *Giardia* parasite. Through immunofluorescence microscopy analysis and chromatin immunoprecipitation (ChIP) studies, they demonstrated its localization in the *Giardia* nucleolus and its interaction with rRNA. Furthermore, by using inhibitors of this enzyme, such as Nicotinamide (NAM), they detected changes in the rRNA levels in the parasite. Based on their findings, these authors propose GdSir2.4 as a potential therapeutic target to combat giardiasis, which deserves further study [67].

The existence of proteins involved in rRNA processing, such as fibrillarin, krr1, snoRNPs and GdSir2.4, as well as the existence of several snoRNAs, were the first indication of the existence of a previously unreported compartment within the nucleus. Furthermore, this suggests that the divergence presented by these proteins could be exploited as a therapeutic target using target-based drug design (Table 2). Finally, the exclusivity of some snoRNAs could be used for gene-silencing therapies.

Subsequently, irrefutable evidence about the existence of a compartment within the nucleus, which would correspond to the nucleolus, was exposed. Which consisted of the precise location of 16S-like and 5.8S rRNA, exclusive proteins of the nucleolus such as fibrillarin, rRNA-pseudouridine synthase (CBF5), and m2,2,7G-cap, as well as the implementation of silver stain techniques specific for NORs, allowed the identification of a small granular structure positioned in the anterior region of both nuclei of this parasite [66,68]. It is worth mentioning that unlike Narcisi et al. [46], these authors cyto-localized the fibrillarin signal within a specific place in both nuclei, possibly because there was better processing to preserve the fine structure of the nucleus, in addition to adequate trophozoite synchronization in interphase [68].

The peculiarities of the *G. lamblia* nucleolus make it a potential therapeutic target for the treatment of giardiasis. It is important to note that the drug targets discussed in this study are essential for gene transcription and have a low mutation rate. Therefore, it would be difficult for the parasite to develop resistance to drugs or therapeutic strategies that target these proteins or non-coding RNAs (ncRNAs).

This is not the first instance in which various therapeutic strategies have targeted the nucleolus, as many genetic diseases in humans have been attributed to this compartment, including multiple types of cancer and, neurodegenerative disorders such as Alzheimer’s and Parkinson’s diseases and even in recent times, this compartment has been associated with aging [69,70,71,72]. There are different therapeutic approaches that could be applied to the treatment of giardiasis, an excellent review of the different approaches to using the nucleolus as a therapeutic target in human diseases is provided by Corman A. et al. in 2023, who emphasize the pharmacological potential of this compartment [72]. One of these is the inhibition of ribosomal biogenesis and maturation by suppressing the activity of RNA polymerase I and other related enzymes. Another approach is the disruption of the interaction between the various components of the RNA polymerase I holoenzyme [70,72,73]. This reduced number of therapeutic approaches regarding the human nucleolus is mainly due to the minimalist nature of the *Giardia* nucleolus.

### 4.2. G. lamblia Nucleolar Proteome

A study conducted in 2020 utilized a bioinformatics analysis to identify the proteome of the *Giardia* nucleolus. The identified nucleolar proteome was then compared with those of representative higher eukaryotes. In this study, the authors summarize that the *Giardia* nucleolar proteome is considerably smaller compared to that of higher eukaryotes; almost 70% of the proteins identified by them correspond to proteins involved in ribosome function and, finally, they identified proteins nucleolar that would be specific to each species. In the case of *G. lamblia*, thirty-nine proteins were unique to this parasite regarding the nucleolar proteome of representative eukaryotes, including humans [74].

It is important to highlight that the thirty-nine proteins are exclusively found in *Giardia*. This data suggests, depending on their essentiality for the parasite, that they could be potential targets for chemotherapy, where the target-based drug design is a promising approach for developing new treatments. Additionally, gene silencing techniques can also be utilized to target their respective mRNAs with minimal off-target effects.

## 5. *G. lamblia* Has a Rudimentary Transcription Initiation Machinery

**Figure 2:**
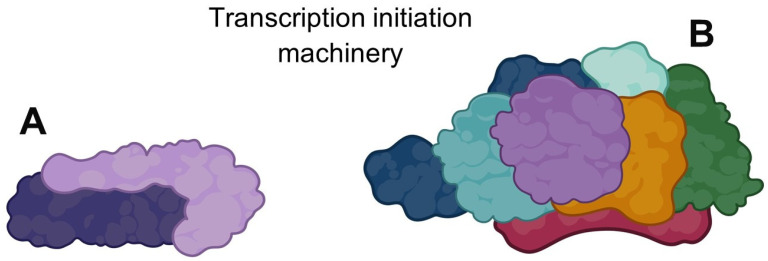
Transcription initiation machinery. (**A**) *G. lamblia*, only two proteins that form the TBP-TAF complex SL1 are reported. (**B**) TBP-TAF complex SL1 of higher eukaryotes, made up of seven proteins. Created with BioRender.com.

Derived from an analysis performed on the genome of *Giardia*, it can be assumed that this parasite has a rudimentary transcription apparatus for all three systems (pol I, II, and III). More specifically, it would have a basic and rudimentary RNAPI transcription initiation apparatus (Figure 2), because of the seven proteins that make up the TBP-TAF complex SL1 in higher eukaryotes; only two have been reported in *G. lamblia* (TBP and RNN3), remembering that this complex oversees the pre-initiation complex formation. Furthermore, only four SL1 complex proteins subunits of the twelve that make up the pre-initiation complex (PIC) in polRNAII have been reported [43,75]. Additionally, it has been reported the existence of eleven orthologs of the fourteen protein subunits that make up the Pol I enzyme complex, as well as two upstream binding factor (UBF) orthologs [75]. Finally, a reduction in the number of amino acids has been reported for several of the components involved in the polRNAI transcription system. [75]. This information suggests that the reduction in the number of components of the different transcription systems, as well as the reduction in the number of amino acid residues that make them up and the divergence that they present regarding human orthologs, make the different protein subunits that make up these systems a starting point for further investigation on whether they can be used as therapeutic targets.

A case to highlight is the TATA Binding Protein (TBP), a universal transcription factor, which has been reported as highly divergent [43]. It is noteworthy that such *G. lamblia* TBP (*Gl*TBP) has mutated three out of four phenylalanine residues, which have been reported as essential in their interaction with the DNA strand, causing through protrusion of their bulky side chains, the bending and the possible opening of the double helix on the promoter region, the latter serves to group the rest of the general transcription factors responsible for forming the pre-initiation complex [43,76,77].

Given the high divergence reported for the universal transcription factor TBP, and because TBP is essential in the transcription of all three types of RNA polymerases, this protein is susceptible to being used as a therapeutic target. This derived from several studies where the sequence of the TBPs of protozoan parasites is compared against that of higher eukaryotes, finding that contrary to what had been reported, the TBPs of lower eukaryotes present a much lower degree of conservation than that reported in higher eukaryotes [78,79,80]. Additionally, the different crystallographic structures of TBPs in complex with other transcription factors reveal that the whole protein is forming interactions with other transcription factors, the so-called TBP-associated factors (TAFs). For example, the characteristic folding of the C-terminal domain of the TBP is a saddle-like structure; such conformation generates two surfaces in the TBP. On the one hand, the convex surface presents interactions with various general transcription factors essential for the assembly of the PIC.

On the other hand, the concave surface interacts with the minor groove of the DNA of the promoter region of the gene to be transcribed [79,80]. Additionally, studies conducted by Santiago et al. [80], reported the first systematic structural analysis of TBPs of parasites, finding many differences between the TBPs of parasites and its counterpart in humans, finding that the convex surface is susceptible to the use of drugs that prevent the proper assembly of the PIC by steric clashes, inhibiting transcription [80]. One aspect to highlight concerning other therapeutic targets is that TBP has an extremely low mutation rate [81]; therefore, the problem of developing resistance to drugs that target this protein would be much more difficult for the pathogen to achieve.

## 6. Drugs under Research for the Giardiasis

Because the nucleolus has become relevant as a chemotherapeutic target for the treatment of different types of cancer and other illnesses [72,73], and due to the recent identification of the *G. lamblia* nucleolus [66,68], some drugs could be repositioned for the treatment of giardiasis. Two potential candidates for drug repositioning are described: Actinomycin D is a cyclic pentapeptide antibiotic that can inhibit the transcription of all three RNA polymerases. This is achieved by intercalating in the double strand of DNA because it presents a high affinity for regions rich in G-C [82], preventing the assembly of the transcription machinery. It is worth noting that *Giardia* rRNA has an unusually high content of G-C [83], which would make this parasite more sensitive to this drug. Experiments conducted by Gaona et al. 2023 (unpublished data) demonstrated a six-fold lower sensitivity to this drug than mammalian cells. Finally, Cisplatin forms covalent adducts, mainly with DNA, although its mode of action is not exactly known. It has been reported that this drug causes a relocation of components of the RNA polymerase I transcription machinery, such as UBF, TBP, and polRNAI holoenzyme [72,84,85,86]. Based on the characteristics of the polRNAI transcription initiation apparatus components, it is plausible that *G. lamblia* could potentially be more vulnerable to this compound; since, as mentioned earlier, *Giardia* exhibits a reduced number of components in its initiation apparatus, these components also show fewer number of amino acids and a higher degree of divergence. Although these drugs have been considered highly toxic, we must not ignore the growing increase in cases of chronic giardiasis, nor the fact that diarrheal diseases, including giardiasis, account for 9% of child deaths worldwide [1,2]. Therefore, its important evaluate whether low doses can eliminate the parasitosis without affecting the host. As mentioned above, actinomycin D exhibits an inhibitory effect at a concentration of six orders lower against *G. lamblia* compared to mammalian cells. In any case, the use of these compounds as a scaffold could lead to the discovery of new compounds that are non-toxic to the host but have the potential to inhibit polRNAI transcription, thereby affecting this parasite with a worldwide distribution.

Finally, we would like to highlight that two drugs are currently undergoing investigation in phases 2 and 3 as potential anti-giardia agents, as documented by the Clinical Trials Information Service (https://www.clinicaltrials.gov/, accessed on 7 August 2023) [87]. The two drugs under evaluation are as follows: Auranofin, which is in phase 2 with the ID NCT02736968. In this study, a daily dosage of 6 mg is being examined over a period of 5 days. Additionally, 20-hydroxyecdysone (an ecdysteroid hormone found in invertebrates) is being assessed. This compound has demonstrated antifilarial effects by inhibiting the filarial ecdysone receptor [88]. This study is in phase 3 and involves the administration of two daily pills, each containing 100 mg, over a span of 10 days, identified by ID NCT04827537. It is important to note that, up to this point, no adverse effects have been reported in patients taking Auranofin, while the results of the study related to 20-hydroxyecdysone have not yet been disclosed.

## 7. Conclusions

In the present review analyzed and provides a historical account of the discovery and evidence supporting of nucleolus of *G. lamblia* as a subnuclear compartment in this parasite. *G. lamblia* has been considered a primitive eukaryote or a highly specialized organism for parasitism. Both hypotheses converge on a limited subset of molecules (proteins and ncRNAs), some of which are exclusive to *Giardia* nucleolus. These subsets of ncRNAs and proteins are seen as an unprecedented opportunity as potential therapeutic targets, considering their participation in ribosome biosynthesis and the essentiality of this pathway for parasite survival.

## Figures and Tables

**Table 1:** Drugs for pharmacological treatment of giardiasis, mechanism of action, and side effects.

Drug	Mechanism of Action	Side Effects [22]
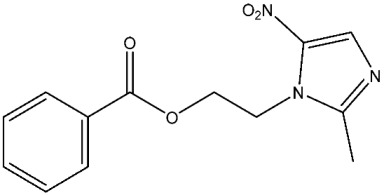 Benzoylmetronidazole	Prevents the production of genetic material. DNA strand rupture. Potential suppression of DNA repair [23,24].	Fever, headache, irritability, lack of coordination, nausea, blurred vision.
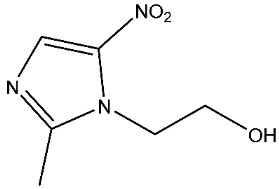 Metronidazole	Prevents the production of genetic material. DNA strand rupture. Potential suppression of DNA repair [23,24].	Fever, headache, irritability, lack of coordination, nausea, blurred vision.
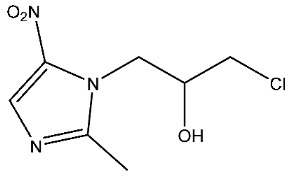 Ornidazole	Prevents the production of genetic material. DNA strand rupture. Potential suppression of DNA repair [23,24].	Fever, headache, irritability, lack of coordination, nausea, blurred vision.
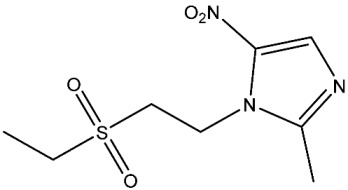 Tinidazole	Prevents the production of genetic material. DNA strand rupture. Potential suppression of DNA repair [23,24].	Bitter taste, metallic taste, difficulty having a bowel movement, dizziness, general feeling of discomfort or illness.
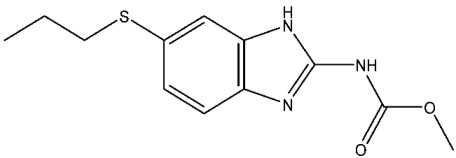 Albendazole	Tubulin assembly suppressor. Fumarate reductase suppressor. VEGF and HIF-1α suppressor. Halt the progression of both the G2 and M phases within the cell cycle [25].	Stomach pain, chest pain, chills, fever.
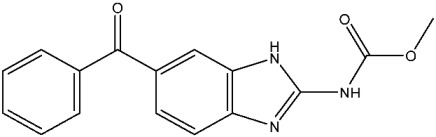 Mebendazole	Tubulin assembly suppressor. Inhibiting glucose absorption [26].	Abdominal or stomach pain or upset, Black, tarry stools, chills.
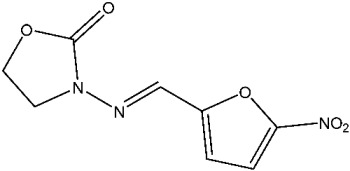 Furazolidone	It is suggested to work through DNA cross-linkage [27].	Joint pain, fever, itching, skin rash or redness, sore throat.
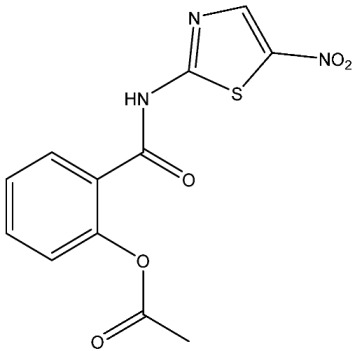 Nitazoxanide	Blocks the activity of pyruvate: ferredoxin/flavodoxin oxidoreductases. Suppression of protein disulfide isomerase activity. Possible binding to nitroreductase GlNR1. Modulation of the expression of heat-shock proteins [28].	Abdominal or stomach pain, headache, nausea, urine changes.

**Table 2:** Proteins and ncRNAs with the potential to be therapeutic targets, suggested therapeutic strategy and function.

Therapeutic Target	Therapeutic Strategy	Function
Fibrillarin	Target-based drug design and gene-silencing therapies.	rRNA processing and maturation.
Krr1	Target-based drug design and gene-silencing therapies.	Assembly 40S ribosomal subunit.
snoRNP (15.5kD Box C/D)	Target-based drug design.	Methylation and Pseudouridylation of rRNA.
GdSir2.4	Target-based drug design.	Deacetylase (modulation rRNA genes expression)
*Gl*TBP	Target-based drug design.	Essential to form PIC.
eIF4E1	Target-based drug design.	Putative methylation and Pseudouridylation of rRNA.
snoRNA (*Gl*sR3, *Gl*sR9, *Gl*sR10 and *Gl*sR11)	Gene-silencing therapies.	Methylation and Pseudouridylation of rRNA.

## Data Availability

Data is contained within the article.
